# Immune regulatory networks coordinated by glycans and glycan-binding proteins in autoimmunity and infection

**DOI:** 10.1038/s41423-023-01074-1

**Published:** 2023-08-15

**Authors:** Salomé S. Pinho, Inês Alves, Joana Gaifem, Gabriel A. Rabinovich

**Affiliations:** 1 i3S – Institute for Research and Innovation in Health, University of Porto, 4200-135 Porto, Portugal; 2 ICBAS-School of Medicine and Biomedical Sciences, University of Porto, 4050-313 Porto, Portugal; 3 Faculty of Medicine, University of Porto, 4200-319 Porto, Portugal; 4 Laboratorio de Glicomedicina, Instituto de Biología y Medicina Experimental (IBYME), Consejo Nacional de Investigaciones Científicas y Técnicas (CONICET), C1428 Ciudad de Buenos Aires, Argentina; 5 Facultad de Ciencias Exactas y Naturales, Universidad de Buenos Aires, C1428 Ciudad de Buenos Aires, Argentina

**Keywords:** Immune response, Inflammation, Infection, Glycans, Glycosylation, Glycan-binding proteins, Translational immunology, Mechanisms of disease

## Abstract

The immune system is coordinated by an intricate network of stimulatory and inhibitory circuits that regulate host responses against endogenous and exogenous insults. Disruption of these safeguard and homeostatic mechanisms can lead to unpredictable inflammatory and autoimmune responses, whereas deficiency of immune stimulatory pathways may orchestrate immunosuppressive programs that contribute to perpetuate chronic infections, but also influence cancer development and progression. Glycans have emerged as essential components of homeostatic circuits, acting as fine-tuners of immunological responses and potential molecular targets for manipulation of immune tolerance and activation in a wide range of pathologic settings. Cell surface glycans, present in cells, tissues and the extracellular matrix, have been proposed to serve as “self-associated molecular patterns” that store structurally relevant biological data. The responsibility of deciphering this information relies on different families of glycan-binding proteins (including galectins, siglecs and C-type lectins) which, upon recognition of specific carbohydrate structures, can recalibrate the magnitude, nature and fate of immune responses. This process is tightly regulated by the diversity of glycan structures and the establishment of multivalent interactions on cell surface receptors and the extracellular matrix. Here we review the spatiotemporal regulation of selected glycan-modifying processes including mannosylation, complex *N*-glycan branching, core 2 *O*-glycan elongation, LacNAc extension, as well as terminal sialylation and fucosylation. Moreover, we illustrate examples that highlight the contribution of these processes to the control of immune responses and their integration with canonical tolerogenic pathways. Finally, we discuss the power of glycans and glycan-binding proteins as a source of immunomodulatory signals that could be leveraged for the treatment of autoimmune inflammation and chronic infection.

## Introduction

The immune system is composed of a network of stimulatory and inhibitory circuits that protects our body from foreign threats or endogenous insults while avoiding immune pathology and destruction of host tissue. Disruption of these molecular checkpoints may lead to a breakdown of self-tolerance and thus to unpredictable inflammatory and autoimmune conditions [1]. On the other hand, interruption of the immune stimulatory pathways may also promote malignancy and sustain chronic infection [1].

Glycans cover all cells and the extracellular matrix with which they interact, serving as major molecular determinants of the fate and function of the immune system. The diversity of glycan structures that emerges at a cell surface stores a tremendous amount of biological information that can be deciphered, at least in part, by glycan-binding proteins (GBPs), including C-type lectins, siglecs and galectins [1]. Multivalent interactions established between lectins and glycans, often called “lattices”, create a spatial interface that integrates the immunological landscape and controls a wide variety of immunopathologic processes, including infection, fibrosis, autoimmunity and cancer [2].

Protein glycosylation is regarded as one of the most common posttranslational modifications of proteins and lipids that, besides providing structural diversity, plays critical roles as specific recognition determinants involved in the communication between cells and the microenvironment, such as the immune system. Immune and stromal cells are equipped with a variety of GBPs devoted to sense and decode the diversity of the cellular glycome. This complementary network of glycans and GBPs are key for pathogen recognition and influence the control of inflammatory and autoreactive responses. Changes in the cellular glycome, occurring in response to genetic and environmental cues, including cytokines, nutrients and hypoxia, are exquisitely sensed by cell surface or soluble GBPs and eventually linked with acquisition of pathologic phenotypes [1, 3–7]. Microorganisms are also covered with glycan-containing molecules, such as bacterial lipopolysaccharides, peptidoglycans, capsular polysaccharides or fungal mannans, among others, which function as pathogen-associated molecular patterns (PAMPs) that are sensed and recognized by GBPs to control infection. The recognition of these glycosylated microbial antigens by the immune system has been exploited for vaccine development [8]. In fact, this microbial glycan-targeting approach has already led to the development of vaccines against type b *Haemophilus influenzae*, *Streptococcus pneumonia* and *Neisseria meningitidis*, as well as others still under research, such as HIV-1, Dengue and Hepatitis C viruses [9].

A remarkable structural variation of glycans occurs in nature, which contributes to biological diversity and consequently to the speciation [10]. Each cell type in individual organisms expresses a distinct array of glycans (a unique glycan signature) that constitutes the “identity card” of a given cell, tissue or organism under defined conditions, and these glycan patterns tend to be conserved within the same species. However, and despite the conservation of genes associated with the glycosylation machinery, there are significant intra- and interspecies variations in glycosylation patterns [10, 11], that place glycans as fundamental molecular pieces for discrimination between self and non-self [1]. The similarity between glycan structures present in host cells and microbes underlies the concept of glycan mimicry [1] and highlights the role of these macromolecules as essential determinants of host-parasite communication. Microorganisms can engage in glycan mimicry to hijack host responses, thus evading immune reactions by decorating themselves with glycans typically found in their hosts, which allow them to establish active infections. Nevertheless, glycan mimicry may also underlie the failure of the immune system to discriminate between self and non-self, an effect that is associated with inadvertent autoreactive responses and autoimmune pathology.

**Fig. 1:**
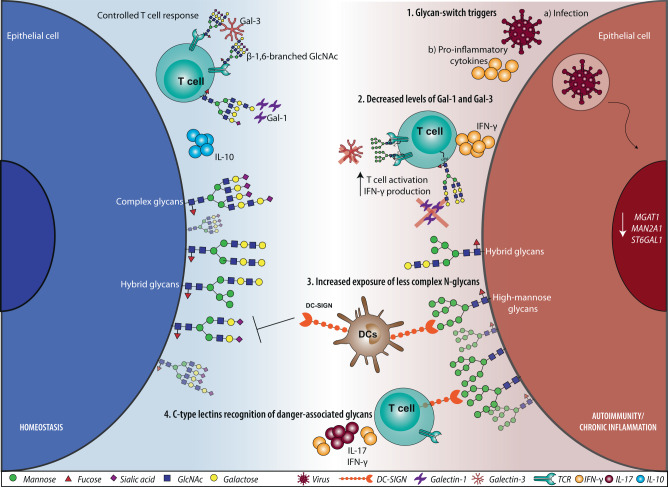
Representation of immune networks mediated by glycans and glycan-binding proteins in autoimmunity and/or chronic inflammation. (1) Environmental triggers, such as microbial infections or the inflammatory microenvironment, may be associated with changes in the cellular glycosylation profile imposed by alterations in the transcription of glycogenes (such as *MGAT1*, *MAN2A1* and *ST6GAL1*). Changes in cellular glycome result in abnormal exposure of altered glycan structures at the cell surface that can be sensed and recognized by specific glycan-binding proteins (GBPs), activating or silencing inflammatory pathways. (2**)** Decreased levels of galectin-1 and galectin-3 were observed in the context of autoimmune diseases, such as rheumatoid arthritis (RA) and systemic lupus erythematosus (SLE). The deficiency of these two GBPs together with changes in the cellular glycosylation profile contribute to perturb the homeostatic glycan-GBP hub, leading to the loss of regulatory/tolerance mechanisms and the consequent activation of pro-inflammatory responses. (3–4) The increased exposure of less complex glycans, such mannose-enriched glycans (3), observed in some autoimmune diseases, is associated with increased recognition by innate immune cells through C-type lectins, such as DC-SIGN, activating immunological pathways and favoring autoimmune pathology (4)

In this review, we will focus on common immune tolerance and homeostatic programs coordinated by glycans and GBPs that control infection and promote resolution of autoimmune and chronic inflammatory processes (Fig. 1).

### Protein glycosylation: structural and functional perspective

Protein glycosylation is a fundamental posttranslational modification that controls essential physiologic processes in cells, tissues and organisms. Most proteins that evolved following appearance of multicellular life are glycosylated, and altered glycosylation is evident in virtually all human diseases [12]. The diverse and complex repertoire of glycans attached to proteins and lipids, collectively termed “the glycome”, guides specific biological processes including protein folding and activity; intra- and intercellular communication, cell developmental processes, and play central roles in host-pathogen interaction and immune system communication [3, 13–15]. The mammalian glycome repertoire is estimated to involve thousands of glycans synthesized by cells and coordinated in diverse structures [3]. Glycan diversity arises from differences in monosaccharide composition (Gal, GalNAc, etc.), linkage between monosaccharide (1–3, 1–4, etc), anomeric state (α *versus* β), branching structures, other substitutions (fucosylation, sialylation, sulfation, etc.) and linkage to their aglycone part (protein or lipid) [11, 16, 17]. This diversity is excelled by a variety of glycan-binding proteins (GBPs) or lectins, including galectins, siglecs and C-type-lectins, mostly expressed by immune cells, providing the basis for deciphering the massive amount of biological information composing the so-called “sugar code” [18]. Understanding the basic principles of protein glycosylation is therefore of utmost importance to basic biology and medicine.

The glycosylation machinery, located in the ER and Golgi compartments, is responsible for assembling glycans diversity through the synchronized action of a portfolio of different glycan-modifying enzymes, including glycosyltransferases and glycosidases. The nature and extent of glycosylation depends essentially on the presence of *N*- or *O*-linked sites in the protein backbone, as well as on the relative abundance of sugar-nucleotide donors and the activity of the glycosyltransferases and glycosidases [19].

*N*-linked glycoproteins are one of the most common forms of protein glycosylation, with up to 90% of glycoproteins being *N*-glycosylated [19]. *N*-glycans are covalently attached to proteins through an amide linkage to the Asparagine (Asn) (N) side chain in the consensus sequence Asn-X-Serine/Threonine, where X is any amino acid except of Proline. The repertoire of *N*-glycans includes an extensive array of mature, complex *N*-glycans comprising sugar additions to the *N*-glycan core, elongation of branching GlcNAc residues by sugar additions of e–g. Galβ1-4GlcNAc (LacNAc) extension, and capping or decoration of elongated branches with e.g. sialic acid or fucose. *N*-glycans are essential in cellular development and homeostasis, as highlighted by the fact that perturbations in their biosynthesis, such as the elimination of the *Mgat1*gene (preventing the synthesis of complex and hybrid *N-*glycans) in mice results in death during embryonic development. Complex *N*-glycans are important in controlling retention of growth factor and cytokine receptors at the cell surface [20], through interactions with GBPs such as galectins. Deletion of genes encoding sialyltransferases, fucosyltransferases, or branching *N-*acetylglucosaminyltranferases that interfere with synthesis of mature *N*-glycans has generally produced viable mice with immunopathology, neuropathology, emphysema, cancer or inflammation [4, 19, 21–25].

Synthesis of *O*-linked glycoproteins involves another type of protein glycosylation that is mainly represented by mucins. In *O*-linked glycoproteins, the addition of carbohydrates, initiated by GalNAc, occurs in serine or threonine residues of a protein. The functions of *O*-glycans depend on their structure, density, as well as on the protein to which they are attached. Cell lines engineered to express altered *O*-glycans, as well as mice with targeted mutations, have revealed the many functions of *O*-GalNAc glycosylation. As an example, a variety of phenotypes have been observed in mice lacking core 1 *O*-glycans in specific tissues including spontaneous colitis, thrombocytopenia, defective lymphocyte homing or alterations in podocyte and renal function, among others [26, 27]. Mice devoid of core 1 and core 2 *O*-glycans specifically in intestinal epithelial cells develop spontaneous inflammation, thus illustrating the critical role of *O*-glycans in gut immunity [19, 25, 28].

Essentially, the biological functions of glycans are associated to three main roles: (a) structural (protein folding, activity and extracellular scaffolds); (b) energy metabolism (serving as a carbon source essential for host-pathogen interactions); and (c) information carriers, that facilitate the communication with the immune system through GBPs. Given the pivotal role of glycans in almost every aspect of cell biology, it is clear that changes in glycan profiles may have a substantial impact in mechanisms of disease pathogenesis, including those operating in infection, chronic inflammation, autoimmunity, and cancer. Moreover, a compelling body of evidence highlights the tremendous potential of glycans and GBPs as valuable diagnostic, predictive and prognostic biomarkers in several clinical applications, some of which will be discussed below.

### Glycans and GBPs as safeguard mechanisms that synchronize immune tolerance pathways

The diverse repertoire of glycans present on the surface of immune cells can be decoded by a set of GBPs and translated into critical immunological responses [13, 29], including: regulation of peptide-loaded major histocompatibility complexes (MHC) [30, 31]; reorganization of T-cell receptor (TCR) and B-cell receptor (BCR) complexes and function [32]; modulation of receptor clustering, stability, endocytosis and signaling [20, 33]; control of immunoglobulins effector functions [34–36]; and activation of danger-associated molecular patterns (DAMPs) and PAMPs [1, 5, 11] crucial for the discrimination between “self” and “non-self”.

**Fig. 2:**
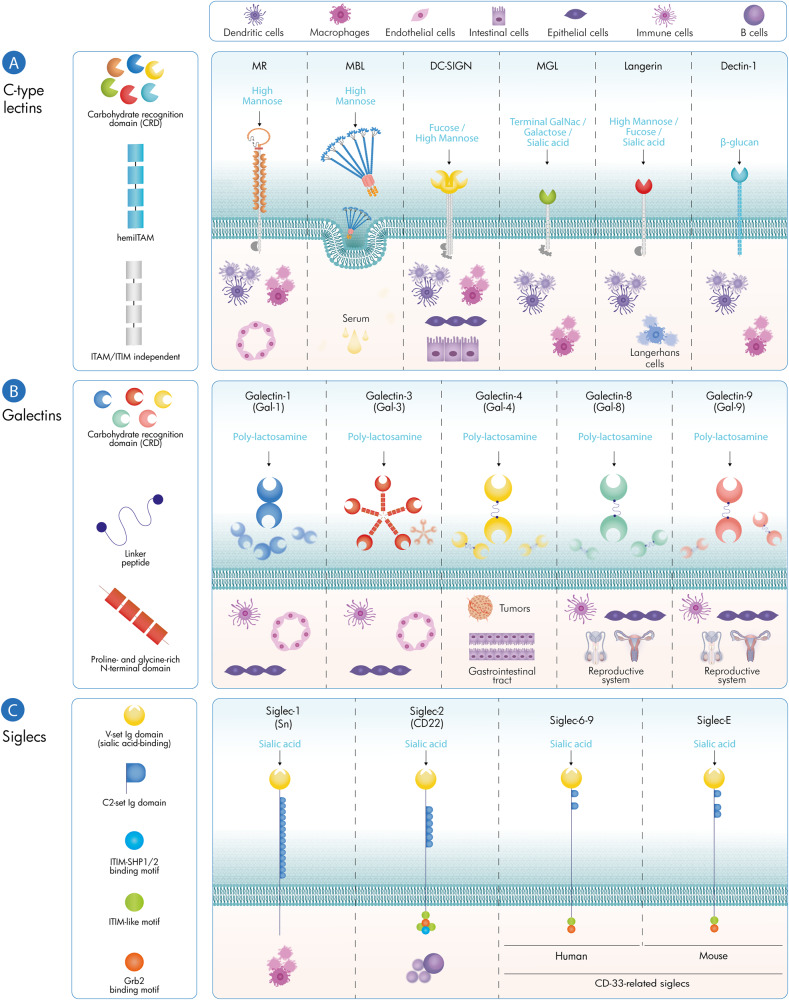
Glycan-binding proteins: main localization and glycans recognition. C-type lectins (**A**) are expressed by a variety of cells and recognize a repertoire of sugar structures associated with either stimulatory or inhibitory responses. Galectins (**B**) are soluble GBPs, which can establish multivalent interactions with glycosylated receptors containing β-galactoside sugars on the surface of multiple cell types. Additionally, siglecs (**C**) are cell surface immune receptors capable of recognizing sialic acid structures

Multivalent interactions between glycans and GBPs have been proposed to act as an essential regulatory hub leading to a plenty of immune-regulatory functions that are vital for immune tolerance. Within the immune system, various classes of GBPs either expressed on immune cells or secreted to the extracellular milieu, are dedicated to recognize specific glycans structures and convey their information into immune-regulatory functions including leukocyte trafficking; pathogen recognition; immune activation, differentiation, and survival [5, 37, 38]. To illustrate this concept, we introduce here the three main lectin families implicated in immune regulation C-type lectins, galectins and siglecs (Fig. 2).

The *C-type lectin receptors (CLRs)* are calcium-dependent GBPs expressed on the surface of many immune cell types, mainly macrophages and dendritic cells (DCs) that predominantly recognize mannose, fucose, *N*-acetylgalactosamine (GalNAc) or *N*-acetylglucosamine (GlcNAc)-containing glycoconjugates [37, 39]. The complexity, spatiotemporal regulation and presentation of glycan epitopes, either monovalent or multivalent, is crucial for selective binding and function of C-type lectins [40]. These include the mannose receptor (MR); DC-specific intercellular adhesion molecule-3-grabbing nonintegrin (DC-SIGN); macrophage galactose-specific lectin (MGL) and langerin [37]. These CLRs can bind to microbial glycans or to altered self-ligands, resulting in cell signaling events that control innate and adaptive immune responses. For example, DC-SIGN can recognize high-mannose glycans and Lewis-type antigens (commonly found in bacteria, fungi and viruses but also in host cells), followed by its internalization and presentation of specific peptides to T cells in the context of MHC II [41, 42].

*Galectins* are a family of evolutionary conserved soluble GBPs that display a variety of regulatory functions on innate and adaptive immunity, mainly by interacting with cell surface glycosylated ligands [6]. Within the immune system, galectins are expressed by virtually all immune cells, being significantly upregulated by activated B and T cells, inflammatory macrophages, decidual natural killer (NK) cells and regulatory T (Treg) cells [5, 43]. Once released through a non-classical ER-Golgi-independent secretory pathway, galectins bind specifically to the disaccharide *N*-acetyllactosamine (LacNAc) structure, presented as multiple units (poly-LacNAc) in complex branched *N*-glycans and core 2 *O*-glycans expressed on cell surface glycoconjugates. These GBPs often form multivalent galectin-glycan complexes on the cell surface—often termed lattices—that play critical roles in endocytosis, segregation and signaling thresholds of glycosylated receptors. A remarkable example of the function of galectins in immunity involves their ability to control the fate and signaling of T cells. The expression of complex branched *N*-glycans on the TCR, catalyzed by the *N*-acetylglucosaminyltransferase-V enzyme (GnT-V; encoded by the *MGAT5* gene) facilitates the elongation with poly-LacNAc structures, which are high-affinity glycosylated ligands for galectins, including galectin-3. Multivalent galectin-3-*N*-glycan interactions result in the formation of cell surface lattices that prevent TCR clustering, increasing the threshold for T-cell activation [32]. Dysregulation of TCR signaling through disruption of complex branched *N*-glycans has significant implications in inflammatory and autoimmune diseases, including autoimmune neuroinflammation and inflammatory bowel disease (IBD; as discussed in detail below). During the activation of homeostatic programs and restoration of immune tolerance, sustained TCR signaling induces upregulation of the *Mgat5* gene leading to GnT-V-mediated glycosylation of the TCR. This phenotype promotes growth arrest of T cells through mechanisms involving an early increase of T-cell activation thresholds which limits TCR clustering via formation of galectin-glycan lattices at sites of immune synapse and, a subsequent increase, at later stages, of cell surface retention of growth inhibitory receptors such as cytotoxic T lymphocyte antigen-4 (CTLA-4) that contributes to T-cell inhibitory activity and activation of homeostatic/tolerogenic pathways [20]. Dysregulation of GnT-V-driven regulatory circuits leads to T-cell hyperactivity and susceptibility to autoimmune diseases, including multiple sclerosis. Furthermore, galectins can also regulate the viability of Th1 and Th17 cell subsets through differential glycosylation of these cells. Evidence demonstrated that Th1- and Th17-differentiated cells express all the glycan profile needed for galectin-1 binding and induction of cell death; however, Th2 cells are protected from galectin-1-induced cell death through differential α2,6-sialylation of cell surface glycoproteins, which prevents galectin-1 recognition and binding [44]. Accordingly, administration of recombinant galectin-1 suppresses chronic inflammation in different models, including experimental autoimmune encephalomyelitis (EAE), collagen-induced arthritis (CIA) and TNBS-induced colitis, by skewing the balance of the immune response towards a Th2-predominant cytokine profile [45]. The regulatory power of GBPs is not limited to T cells, having also a key role in B-cell development and maturation in which stromal cell derived-galectin-1 may serve as a ligand for pre-BCR signaling [46]. In this regard, recent evidence highlights the critical role of galectin-9 in setting the threshold of B-cell activation, by regulating the interactions between the BCR and Toll-like receptors (TLRs); loss of this regulatory network was associated with development of autoimmunity [47]. Similar to T cells, GnT-V-mediated *N*-glycan branching has also been shown to promote positive selection of B cells by increasing pre-BCR/BCR signaling via CD19 surface retention. Branching deficiency reduced surface expression of the pre-BCR/BCR co-receptor CD19 and promoted spontaneous death of pre-B cells and immature B cells in vitro [48]. Additionally, galectins can also shape the myeloid cell compartment. Whereas galectin-1 favors induction of IL-27-producing tolerogenic dendritic cells and promotes induction of M2-type macrophages and microglia, galectin-9 induces the expansion of myeloid-derived suppressor cells [49–51]. Thus, galectin-glycan interactions can reprogram the fate and function of lymphoid and myeloid cells by activating diverse tolerogenic pathways. However, in spite of this evidence, galectins may also play pro-inflammatory roles in a variety of pathologic contexts [52]. To illustrate this concept, galectin-1 has been shown to function as a danger-associated molecular partner (DAMP) or alarmin that amplifies the inflammatory response during sepsis [53]. Moreover, galectin-3 has shown broad pro-inflammatory activities by modulating survival, differentiation, recruitment and effector functions of neutrophils, mast cells, macrophages and T cells [54, 55]. Additionally, galectin-4 can sustain signaling of pro-inflammatory cytokines including IL-6 during intestinal inflammation [56]. Thus, the synchronized action of different members of the galectin family providing either anti- or pro-inflammatory signals at early or late stages of immune responses may contribute to orchestrate the inflammation cascade of different pathophysiologic settings. Furthermore, in addition to their extracellular functions, galectins may also exert intracellular functions during ongoing inflammatory responses [57]. In fact, in response to a variety of challenges, intracellular galectins, including galectin-3 and -8 can accumulate and bind to host glycans displayed on damaged endocytic vesicles and trigger intracellular defense mechanisms, such as autophagy [58–60]. Moreover, galectins can also act as key intracellular effectors of immune cell apoptosis, cell cycle regulation, cell signaling and pre-mRNA splicing [60], suggesting that this highly conserved GBPs may influence immune cell homeostasis through both extracellular and intracellular mechanisms.

*Siglecs* (sialic acid-binding immunoglobulin-like lectins) are a family of transmembrane proteins with variable numbers of immunoglobulin domains that are widely expressed in neutrophils, monocytes, B cells, DCs, NK cells, eosinophils and basophils and are barely detected in T cells [37, 38, 61]. These GBPs specifically recognize sialic-acid-containing glycans expressed on cell surface receptors linked to immunoregulatory sequences (either immunoreceptor tyrosine-based inhibitory motifs or immunoreceptor tyrosine-based activation motifs) [61]. Siglec-1 (sialoadhesin or CD169) is a major receptor expressed on macrophages which plays a key role in both self-and pathogen recognition. Relevant human pathogens, such as *Campylobacter jejuni, Escherichia coli*, *H. influenza*, *Neisseria meningitides*, group B *Streptococcus*, among others, express sialic acid-containing glycoconjugates that are recognized by Siglec-1 on macrophages as a mechanism to control phagocytosis, clearance and antigenic presentation of pathogenic bacteria [62]. Siglec-2 (also known as CD22) is mostly expressed on B cells, modulating B-cell receptor (BCR) signaling, and also dendritic cells [61]. Siglec-2 tends to recognize sialic acid ligands generated by α2,6-sialyltransferase 1 (ST6GAL1) in neighboring CD22 molecules, forming homotypic multimers in *cis* that preclude the interaction of CD22 with BCR in trans, an effect associated with changes in the threshold of B-cell activation. Loss of CD22 sialylated ligands (due to *ST6GAL1* deficiency) leads to endocytosis of BCR and decreased BCR activation [63]. Interestingly, Siglec E has also been shown to play an important inhibitory role in host-pathogen interactions. As an example, the Group B Streptococcus (GBS) surface capsule containing sialic acids engage Siglec E on leukocytes to blunt NF-κB and MAPK activation.It was demonstrated that upon GBS infection, *Siglec-E*^*−/−*^ macrophages displayed increased pro-inflammatory cytokine secretion, phagocytic rate and bactericidal activity against pathogens [64]. Also, human Siglec-9 was shown to bind high molecular weight hyaluronan (HMW-HA), a glycan that is present in the capsule of pathogen group A *Streptococcus*. This was found to subsequently limit neutrophil extracellular trap (NET) formation, oxidative burst, and apoptosis [65]. This sialic acid-siglec mimicry effect that characterizes some host-parasite relationships constitutes a way of influencing host innate immune responses and shaping the course of infection.

In *antibody-mediated immune response*, the glycan-GBPs hub also plays an important role. Immunoglobulins and most of components of the complement system are highly glycosylated. IgG antibodies are the predominant antibody class in tissues and circulation, being key effectors of the humoral immune response by triggering leukocyte activation and inflammation. All IgG Fc domains contain a single, highly conserved, glycosylation site in Asn297 that carries complex *N*-glycans. Over 30 different glycan variations were detected on circulating IgG in healthy individuals, which reflects a significant heterogeneity in the IgG Fc glycome [34]. In fact, the type of glycans attached to the Fc region of IgG regulates binding to Fc gamma receptors (FcγRs), instructing either a pro- or an anti-inflammatory response. As an example, terminal sialylation of Fc glycans modulates FcγR binding. The presence of α2,6-linked sialic acid on Fc glycans significantly reduced FcγR affinity being associated with anti-inflammatory activity. Strikingly, glycoengineering of intravenous immunoglobulins (IVIgs) leading to increased Fc sialylation resulted in a 100-fold increase of their anti-inflammatory activity in a mouse model of arthritis [66]. The anti-inflammatory properties of IgG Fc sialylated *N*-glycans were found to be mediated by enhanced binding of sialylated IgG to DC-SIGN, triggering the expression of immunosuppressive cytokines [67], although other studies demonstrated anti-inflammatory effects of IVIgs that were independent of sialylation and DC-SIGN expression [68]. On the other hand, IgG Fc hypergalactosylation has been consistently described to display enhanced anti-inflammatory activities and mouse studies suggested that Fc galactosylation promotes the association between FcγRIIb and the C-type lectin receptor Dectin-1 as well as galectin-3 which in turns inhibits C5a-mediated inflammatory responses [69, 70]. Conversely, agalactosylated IgG Fc has been associated with pro-inflammatory activity in many autoimmune diseases including IBD [71] and infectious processes such as COVID-19 [36]. Terminal agalactosylation on IgG1, observed in patients with rheumatoid arthritis, has been demonstrated to appear in the circulation preceding the disease onset [72, 73]. The pro-inflammatory effects of agalactosylated IgG Fc was associated with its binding to the mannose-binding lectin MBL, allowing activation of the lectin complement pathway [74].

Interestingly, another example that supports the relevance of glycosylation in immunoglobulin biology relates to IgE.This immunoglobulin s essential for initiation of allergic reactions being the least prevalent immunoglobulin in circulation. Results showed that genetic abrogation of oligomannose glycans in IgE Fc impact on IgE secondary structure, impairing the binding to FcεRI and consequently hampering IgE-driven mast cell degranulation, thus preventing anaphylaxis [75]. This biological impact of IgE glycosylation was also demonstrated in vivo. IgE lacking N384 glycans were unable to initiate immediate anaphylaxis in a passive cutaneous anaphylaxis mouse model [75].

Taken together, the glycan-GBPs hub provides a new layer of immune regulation that governs both innate and adaptive immune responses. Changes in glycosylation are often a hallmark of the transition from normal to inflammatory states, and the glycan-GBPs hub is a master tuner of this transition, providing new opportunities for diagnostic and therapeutic interventions based on the underlying glycobiology [76].

### Glycans and GBP in infection: from viruses to bacteria, fungi and parasites

Host-pathogen interactions involve a sequential number of events, involving diverse cellular and molecular interactions, including those mediated by glycans and GBPs [77]. Differently from pathogen template-driven epitopes (such as proteins and DNA), microbial glycans constitute one of the most immediate pathways of self/non-self discrimination [1]. However, the specific mechanisms that control interactions between the pathogen’s glycome and the host-derived GBPs have not been fully elucidated. In this regard, the host immune system is equipped with a myriad of GBPs capable of identifying a pathogen that is mounting an innate or adaptive immune response. Accordingly, glycans have been proposed to control the balance between commensal and pathogenic microbial populations and have been shown to be key determinants in the establishment of acute or chronic infections [1, 78].

As eukaryotic cells, *fungi* are well equipped with components of the ER and Golgi glycosylation machinery, resulting in an *N*-glycome with structures that are similar to those found in mammalian cells. Fungi *N*-glycosylation is often restricted to high-mannose type glycans with extensive elongation with α-1,6-linked mannose, mannan (linear β1–4-linked mannose) and β-glucan (linear β1–4- and β1–3-linked glucose). *O*-glycans are also abundant in the fungal cell wall, specifically Ser/Thr-linked *O*-mannose glycans. This type of glycosylation is mediated by protein O-mannosyltransferases (PMTs), homologous to mammalian POMTs. Fungal *O*-glycans are mostly involved in adhesion processes, whereas *N*-glycans play a major role in host recognition [1, 79–81]. The specific glycan structures expressed in fungi are recognized by host GBPs such as DC-SIGN, MR, Dectin-1 and -2, and galectins [82, 83], that contribute to tailor innate and adaptive immune responses. To illustrate this concept, during *Candida albicans* infection, DC-SIGN-mediated glycan recognition leads to the activation of TLR signaling on human DCs, resulting in elevated IL-10 production [84]. On the other hand, fungal-derived mannosylated glycans can induce a pro-inflammatory response through its recognition by MR expressed on macrophages, leading to development of Th17 responses [85].

Dectin-1 and 2, transmembrane GBPs expressed mainly by DCs and macrophages, bear an intracellular immunoreceptor tyrosine-based activation motif (ITAM), that upon ligand recognition on fungi, activates the caspase recruitment domain family member 9 (CARD9)-nuclear factor-κB (NF-κB) axis, resulting in the transcription of various pro-inflammatory cytokine genes. Despite recognizing different glycans (Dectin-1 recognizes β-glucans, whereas Dectin-2 binds to α-mannans) both GBPs are implicated in host responses against *C.albicans* and other similar fungi. Dectin-1 often synergizes with TLRs and DC-SIGN to allow cell signaling and synthesis of pro-inflammatory cytokines [86, 87]. Through a similar mechanism, Dectin-2 activation may be associated with Th17 cell polarization [88, 89].

Galectins are also implicated in fungi recognition [77]. Extracellular galectins are often associated with trapping and encapsulation of several pathogens, whereas intracellularly they function as part of the autophagy machinery as has been noted above. Interestingly, galectin-3, but not galectin-1, has been shown to bind to β1,2-linked oligomannosides, resulting in the elimination of *C. albicans* [90, 91].

The glycosylation of *viruses* relies on the activity of host enzymes linked to the glycosylation machinery, which contribute to create similar glycans as those found in infected cells. The capacity of a virus to replicate and change *N*-glycosylation patterns impacts on their ability to be recognized by the immune system, infect new cell types and evade immune responses. As an example, the envelope glycoproteins of certain viruses, including influenza A and HIV are highly covered by mannosylated clusters. This glycan shield confers many advantages to the virus, by hiding epitopes from immune surveillance but also for attachment to host cells, signaling and immune cell activation [92]. In this regard, HIV virus glycoproteins are highly decorated with *N*-glycans, mostly high-mannose but also complex and hybrid type *N*-glycans [93]. Interestingly, the vulnerability site for antibody neutralization involves areas surrounded by high-mannose N-glycan shields [94, 95]. On the other hand, SARS-CoV-2, the virus responsible for COVID-19, is also decorated with glycan moieties [96, 97]. The Spike protein contains 22 *N*-glycosylation sites, which are important for cellular anchoring and colonization of endothelial cells through DC-SIGN recognition [98]. Strikingly, this process may be reverted through the use of glycomimetics [99, 100]. The incubation of a mannose-based multivalent glycopolymer with DC-SIGN-expressing cells leads to inhibition of SARS-CoV-2 Spike protein binding, and prevention of DC-SIGN-mediated trans-infection of angiotensin converting enzyme-2 (ACE)+ cells [99]. A different mannose-based glycomimetic, Polyman26, already known to inhibit DC-SIGN-mediated HIV infection of CD8^+ ^T cells, was also able to inhibit Spike protein binding to DC-SIGN, leading to a decrease in trans-infection of DC-SIGN-expressing cells [100]. Accordingly, greater DC-SIGN expression on peripheral monocytes correlated with better prognosis of COVID-19 patients, which was also associated with higher production of pro-inflammatory cytokines [101, 102].

Although *O*- and *N*-glycosylation processes are widely described in eukaryotes, these mechanisms are also found in prokaryotes, such as *bacteria*. Flagellins and adhesins are *O*-glycosylated proteins that have a critical role on bacterial flagellin and pili function [103, 104]. *Mycobacterium tuberculosis* (Mtb) is a typical example of a bacteria in which glycans are instrumental for infection. Mtb is covered by a dense layer of polysaccharides crucial for internalization, colonization, and subsequent infection, being a well-studied example of *O*-mannosylation [105, 106]. Mannose-capped lipoarabinomannan (ManLAM), a macroamphiphilic lipoglycan present on the surface of Mtb cell envelope [107], is recognized by several C-type lectin receptors present on macrophages and DCs, such as MR, DC-SIGN and Dectin-2 [108–110]. The recognition of Mtb glycans may shape immune responses by impairing phagosome maturation in macrophages, suppressing DC maturation and function and promoting CD4^+^ T-cell activation [109, 111]. Indeed, bacterial protein *O*-mannosylating machinery functions as a critical virulence factor, as demonstrated by inactivation of genes encoding protein *O*-mannosyltransferases, including *Rv1002c*, in Mtb and *MSMEG_5447* in *Mycobacterium smegmatis*, which led to severe deficiencies in in vitro bacterial growth and reduced lysozyme and acidic stress, as well as decreased virulence in vivo [112, 113]. These findings reinforce the key role of glycans in *Mycobacteria*-driven immune evasion mechanisms. Further studies focused on mycobacterial glycosylation processes and its impact on host-pathogen dynamics are essential for tailoring new strategies to tackle Mtb infection.

Additionally, certain bacteria express sialic acid on their surface using this saccharide as an energy source. Interestingly, they develop multiple strategies for sialic acid uptake, including the synthesis of sialidases (or neuraminidases) which removes sialic acid from complex glycan structures of host cells. Since some type of bacteria are not equipped with mechanisms to bind sialic acid, sialidases are essential for removing this monosaccharide and exposing underlying glycan-binding sites for pathogens [114]. CMP-Sia, the activated form of sialic acid, can be utilized by bacteria, such as *Neisseria gonorrhoeae*, promoting evasion of complement-mediated killing. This is mostly due to increased incorporation of *N*-acetylneuraminic acid (Neu5Ac; predominant form of sialic acid) to the cell surface, leading to increased factor H and subsequent inactivation of the classical complement pathway [115]. Furthermore, several bacteria use sialic acid as a mechanism to evade host immune response, as a molecular mimicry program [116].

Galectins have emerged as important players in host immunity. In fact, galectin-4 and galectin-8 have been demonstrated to bind and kill *E. coli* strains by recognizing bacterial surface glycans, mimicking blood group antigens [117]. Galectin-4 binding to bacterial surfaces is also described to limit intracellular bacterial motility, inducing inflammasome activation in infected intestinal epithelial cells, which reinforces the key role of galectins in host-gut microbe interactions [118]. Interestingly, galectin-3 was also reported to recognize a myriad of microbial glycans and display antimicrobial activity against *Providentia alcalifaciens* and *Klebsiella pneumoniae* strains [119], which highlights the crucial impact of galectins in bacterial infection settings and in tailoring effective immune response against bacterial pathogens. In fact, galectin-1 has been demonstrated to bridge glycans present on the surface of *Chlamidia trachomatis* and epithelial cells, thus enhancing bacterial infection in vitro and in vivo [120]. Likewise, this lectin has been shown to function as a receptor for the sexually transmitted human parasite *Trichomonas vaginalis* [121]. On the other hand, galectin-1 can be co-opted by different pathogens, including the bacteria *Yersinia enterocolitica* and the parasite *Trypanosoma cruzi*, to generate tolerogenic circuits and evade immune responses [122, 123]. Both galectin-1 and galectin-9 have been proposed to reprogram viral latency during HIV-1 infection [124, 125]. Furthermore, galectin-1 prevents cell fusion induced by Nipah virus envelope glycoproteins and enhance DC secretion of pro-inflammatory cytokines [126]. Finally, upon *E. coli* infection and intracellular LPS sensing, galectin-1 can be released through mechanisms involving non-classical inflammasome activation, gasdermin D cleavage and pyroptosis, acting as a danger-associated molecular pattern (DAMP) or alarmin during sepsis [53].

Similar to *O*-glycosylation, *N*-glycosylation is also found in bacterial species. One of the most well-studied *N*-glycosylation processes in bacteria and, in fact, the first to be identified is in *C. jejuni*, the major causative agent of human bacterial diarrhea worldwide. Protein *N*-glycosylation in *C. jejuni *has been described to alter its pathogenicity. Moreover, a similar *N*-glycosylation pattern is also found in other bacterial species, such as *E. coli* [127]. *C. jejuni N*-linked glycosylation was described to promote bacterial fitness in the intestine [128]. Mutations in genes associated to the *N*-linked protein glycosylation system (Pgl) are associated to prevention of colonization ability to invade intestinal epithelial cells [129]. Interestingly, *N*-linked glycosylation partners encoded by the *Pgl* locus can alter *C. jejuni *proteins, enabling them to bind the macrophage galactose-type lectin (MGL) [130], supporting its immunomodulatory properties. Thus, an integrated network of glycans and GBPs can mediate infection, invasion and immune evasion programs displayed by a wide variety of intracellular and extracellular pathogens.

### Glycans and GBPs in chronic inflammation and autoimmunity

The transition from healthy toward an inflamed tissue is often accompanied with alterations in cellular glycosylation. In the context of inflammatory and autoimmune diseases, the pro-inflammatory cytokine milieu can promote significant changes in cellular glycosylation, including downregulation of *N*-acetylglucosaminyltransferase V (*MGAT5*) expression.

*Inflammatory bowel disease (IBD)* is a chronic, debilitating disorder of the gastrointestinal tract, whose etiology remains to be fully clarified. Glycans have emerged as pivotal players that control the transition from healthy to inflamed intestine with several glycosylation abnormalities being described in IBD. As an example, mice lacking β1,6-branched *N*-glycans (*Mgat5*^−/−^) display increased susceptibility to severe forms of colitis [32]. Treatment of *Mgat5*^−/−^ mice with GlcNAc was shown to significantly reduce disease severity and progression [22].

Glycosylation is also known to regulate the activity of GBPs, including galectins during autoimmune inflammation. As an example, a glycosylation-dependent galectin-1-driven circuit has been shown to control intestinal homeostasis preventing the development of IBD. Notably, upregulation of *ST6GAL1* mRNA, in colonic biopsies from IBD patients, was correlated with interruption of galectin-1 signaling, whereas higher *C2GNT1* mRNA levels were positively correlated with galectin-1 binding and suppression of intestinal inflammation, highlighting an alternative mechanism through which glycosylation shapes gut immunity [25]. Likewise, in IBD, defects in *N*-glycan branching were found in mucosal T cells from patients with ulcerative colitis (UC), an effect that was associated with T-cell hyperactivation and increased disease severity [23]. Indeed, β1,6-branched complex *N*-glycans have been shown to influence TCR signaling, serving as ligands for galectin-3 recognition with subsequent impact on the threshold of T-cell activation and development of autoimmune diseases [32]. Ex vivo supplementation of T cells from UC patients with GlcNAc promoted branched *N*-glycans in the TCR leading to suppression of pro-inflammatory responses and control of T-cell activity [22].Additionally, *LGALS9*, a gene that encodes for human galectin-9, was also found to be associated with IBD. *LGALS9*, has been linked to 1 of nearly 30 loci associated with IBD in genome-wide association studies [131]. In mice, galectin-9 was shown to be essential for the regulation of cellular proliferation and epithelial restitution after intestinal epithelial injury. Accordingly, *Lgals9−/−* mice showed increased susceptibility to severe forms of colitis [132]. Thus, galectin-9 functions as a key regulator of epithelial cell proliferation and intestinal mucosal homeostasis.

Additionally, alterations in fucosylation were also described in IBD. The *FUT2* gene, encoding for the α1,2-fucosyltransferase enzyme, is known to control the expression of ABO blood group antigens on the gastrointestinal mucosa. Individuals bearing polymorphisms in *FUT2* are defined as “non-secretors”, a phenotype demonstrated to be associated with increased susceptibility to Crohn’s disease (CD) [133]. Interestingly, intestinal commensal microorganisms promoted *Fut2* gene expression in mice, leading to increased α1,2-fucose, in a mechanism dependent of the activation of type 3 innate lymphoid cells (ILC3) and IL-22 [134]. Some commensals such as *Bacteroides fragilis* were able to salvage free fucose and utilize it for its own glycoconjugates, in a possible molecular mimicry mechanism, with fitness advantage in colonization and evasion from protective immune response [135]. Besides, gut bacterial species are equipped with mucinases that allows them to utilize mucin as carbon source. It was revealed recently that bacterial mucinases can adapt to the diverse composition and patterns of O-glycans of mucins, as a conserved mechanism displayed by *Akkermansia muciniphila* and *Bacteroides thetaiotaomicron *strains [136]. Nevertheless, this symbiotic relationship may be impaired by external cues, such as antibiotics treatments. For instance, it was recently described that mice treated with carbapenems (broad-spectrum antibiotics) have an expansion of *B. thetaiotaomicron*. This abnormal expansion leads to an increased expression of enzymes associated with the degradation of mucin glycans that aggravates colonic graft-versus-host-disease (GVHD) [137].

C-type lectins are also seminal players in maintaining intestinal homeostasis. For instance, DC-SIGN expression was found to be increased in intestinal epithelial cells from both CD and UC patients and correlated with disease severity [138]. Consistently, the same was observed in mouse models of colitis, with impact in gut immunity, showing that, during IBD, intestinal epithelial cells can modulate tissue-associated immune populations via DC-SIGN signaling [138]. Also, mice lacking Dectin-1 show increased susceptibility for chemically-induced colitis due to their lack of capacity to respond to fungal species [139]. These findings reinforce the paramount collaborative role of microbiota, mucosal immunity and glycosylation in shaping intestinal homeostasis.

Gastrointestinal inflammation can also be triggered by infection with bacterial pathogens. Recurrent human food-poisoning by *Salmonella enterica Typhimurium* was shown to induce chronic intestinal inflammation in mice, leading to colitis onset, with mammalian Neu3 neuraminidase as the responsible factor for intestinal alkaline phosphatase desialylation and deficiency. Interestingly, abrogation of Neu3 prevented the development of severe colitis [140]. In this regard, infection with *Helicobacter pylori* increased the expression of α1,4-*N*-acetylglucosaminyltransferase (α4GnT) in the gastric mucosa, promoting bacterial colonization and subsequent establishment of chronic inflammation [141].

During the resolution of autoimmune inflammation, glycans have been demonstrated to enhance tolerogenic circuits and restore immune cell homeostasis through different mechanisms, including immune cell receptor recognition and signaling. Nearly all cell surface receptors are glycosylated including the signaling receptors TCR and BCR, the activation markers CD25 and CD69, immune checkpoints molecules such as CTLA-4, PD-1 and TIM-3 and cell adhesion molecules such as cadherins and integrins, among others [142]. This additional layer of complexity finely tunes receptor recognition, binding and function, which may be altered in the context of disease. Particularly in *multiple sclerosis* (MS), an autoimmune disease characterized by neuroinflammation and demyelination, loss of β1,6-branched *N*-glycans in the TCR molecule was associated with hyperactivity of T cells. In fact, TCR threshold is highly regulated by the formation of lattices between complex branched *N*-glycans and galectin-3, and disruption of this physical constrain results in a facilitated TCR clustering and T-cell activation. *Mgat5*^−/−^ mice, devoid of β1,6-GlcNAc branched *N*-glycans, showed increased susceptibility to experimental autoimmune encephalomyelitis (EAE) [32], a mouse model of MS. More recently, the role of branched *N*-glycans in controlling B-cell responses was also highlighted; loss of complex *N*-glycans in B cells led to a switched cell phenotype towards a more pro-inflammatory profile, in which surface retention of CD19 and BCR receptors was observed [143]. In vivo B-cell-specific ablation of *Mgat2* led to greater susceptibility to EAE. Collectively, these evidence, including T or B-cell glyco-reprograming, pinpoint the relevance of *N*-glycan branching in fine-tuning and controlling cell activation in an autoimmune context. One of the well-established mechanisms implicated with branching-mediated control of immune response is the multivalent interaction with galectins. In fact, mice devoid of galectin-1 (*Lgals1*^*−/−*^) showed increased Th1 and Th17 pro-inflammatory responses, enhanced microglia activation and higher susceptibility to EAE [44, 49, 50], thus emphasizing the pro-resolving and anti-inflammatory activity of this GBP. On the contrary, mice lacking galectin-3 (*Lgals3*^−/−^) showed reduced EAE severity, highlighting the pro-inflammatory role of this endogenous lectin. One of the proposed mechanisms involve modulation of the DC compartment, since *Lgals3*^−/−^ DCs inhibit the production of IL-17 when co-cultured with T cells, suggesting that this chimera-type lectin exacerbates demyelinating disease by augmenting IL-17 and IFN-γ synthesis and decreasing IL-10 production [144].

*Rheumatoid arthritis (RA)* is one of the most prevalent autoimmune diseases worldwide, characterized by chronic inflammation of the joints. The pathogenesis of RA has been widely associated with alterations in the glycome either in IgG as well as other glycoproteins present on immune cells and synovial fibroblasts. Specifically, a recent study has demonstrated that tumor necrosis factor (TNF)-mediated inflammation is associated with a reduction in α2,6-sialylation and increased extension of LacNAc structures in synovial fibroblasts, leading to a higher pro-inflammatory profile characterized by enhanced production of IL-6 and CCL2. Loss of α2,6-sialylation facilitated galectin-3 binding and instruction of a pro-inflammatory response [145, 146]. Concomitantly, TNF-α successfully upregulated the expression of β1,4-GalT-I, an enzyme responsible for the addition of a galactose residue to the terminal *N*-acetylglucosamine [147]. Thus, concurrent with a reduction of α2,6-sialylation, TNF-driven mechanisms are also associated with unmasking of galectin-3-binding sites on synoviocytes from RA patients. Interestingly, early studies demonstrated that protein administration or genetic delivery of galectin-1 suppresses the arthritogenic process by skewing the balance toward a Th2 phenotype [148]. Interestingly, galectin-1 levels increase with the severity of the disease in plasma from patients with RA whereas circulating galectin-3 levels were downregulated, suggesting that a cross-talk between these two lectins may regulate the exacerbation and resolution of an inflammatory response [149].

It has been observed in RA that loss of IgG sialylation, in inflamed joints, contributes to pro-inflammatory effects of antibody-driven immune responses. In fact, the anti-inflammatory activity of IgG was attributed to sialylation of its Fc glycan. In an attempt to recover IgG sialylation profile and to prevent development of inflammation, Pagan *et al* developed a soluble sialyltransferase (*ST6Gal1*), which promoted IgG sialylation in vivo. The exogenous sialylation of pathogenic IgG was shown to be effective in preventing the activation of Fcγ receptor, namely through the binding of sialylated Fc to DC-SIGN, resulting in increased surface expression of the inhibitory FcγRIIB on inflammatory effector cells, thus promoting a Th2-STAT6-IL-4 axis [150]. These findings underscored the therapeutic potential of IgG glycoengineering in vivo.

Patients with *type 1 diabetes* have also found to harbor a non-secretor FUT2 phenotype, being unable of express blood group antigens which may be associated with disease susceptibility. In turn, this phenotype can also lead to increased resistance to pathogen infections [151], highlighting the complex dynamics between controlled and exacerbated immune responses in host protective responses and autoimmune inflammation. Furthermore, changes in glucose metabolism in type 1 diabetes have been shown to impact the activities of C-type lectins, such as MBL, which expression is associated with increased risk of disease complications, such as diabetic nephropathy [152, 153]. Galectins have also been reported to be play roles during inflammatory processes underlying metabolic disorders, such as diabetes and obesity. Interestingly, galectin-1 and galectin-9 upregulation or their administration in animal models has been demonstrated as a protective strategy against the development of type 1 diabetes [154]. Moreover, recent studies identified a role for galectin-1 in regulating glucose metabolism through modulation of pancreatic insulin secretion, highlighting novel opportunities to control type-2 diabetes as well [155].

*Systemic Lupus Erythematosus (SLE)* is another example of autoimmune disease in which glycosylation seems to play an important role in breaking self-tolerance. An extensive 2D characterization of the kidney glycome from lupus nephritis patients showed an enrichment of high-mannose structures in detriment to complex and branched *N*-glycans. This was shown to be a result of downregulation of *MAN2A1*, a gene encoding the mannosidase I, responsible for hydrolysis of the second mannose antennae and the progression of the *N*-glycosylation pathway towards complex *N*-glycans. In this regard, an increased expression of mannosyltransferases gene (*POMT1*) was observed, resulting in an abnormal exposure of *O*-mannose structures. Interestingly, this unique glycosylation signature was shown to be specific of SLE and not derived from other kidney diseases [156]. Accordingly, an early study reported a case of two sisters with α-mannosidosis (a rare genetic condition characterized by the deficiency of mannosidase enzyme) who both developed SLE [157]. Mouse models have also helped to establish tissue glycome changes as a central hallmark of SLE. As an example, mannosidase II null mice spontaneously developed lupus disease, with signs of glomerulonephritis, C3 kidney deposition, and high levels of autoantibodies, all features observed in human lupus disease [158, 159]. Moreover, *Mgat5*^−/−^ mice developed spontaneous glomerulonephritis with aging, a feature also found in lupus patients [32]. In this line, analysis of the glycome of kidney cells from a lupus mouse model, *MRL-lpr*, a lower abundance of complex *N*-glycans together with an accumulation of paucimannose structures were identified [160]. Altogether, these observations converge in a specific glycosylation signature found in lupus kidney that may be tuning self-recognition. In fact, abnormal high-mannose *N*-glycans found in human kidney tissue were found to be recognized by DC-SIGN, potentially instructing a pro-inflammatory response [156]. In fact, not only DC-SIGN but also the MR and MBL were found to be abundantly expressed in lupus renal tissue. Thus, recognition of aberrant glycoepitopes by GBPs appear to be critical for driving pathogenic responses during during disease progression which can be abrogated by treatment of anti-DC-SIGN monoclonal antibodies [159, 161, 162].

An early component of the immunopathogenesis of SLE is the hyperactivation of T cells. The ratio between sialyltransferases and neuraminidases (*ST6GAL1/NEU1* and *ST3GAL6/NEU1*), which describes the terminal sialylation levels, was found to be increased in T cells from SLE patients. This effect resulted in decreased galectin-1 binding [163], which was recovered after neuraminidase treatment. Since galectin-1 binding to T cells is often associated with an apoptotic signal [164], it is highly relevant to associate loss of galectin-1 binding with resistance to T-cell death in SLE. In this regard, mice lacking galectin-1 developed a spontaneous autoimmune disease during aging that recapitulates the clinical signs of Sjögren disease and SLE [165]. Likewise, galectin-3-deficient mice develop a SLE-like disease with spontaneous formation of germinal centers via IFN-γ-dependent mechanisms [166]. Finally, although the role of galectin-9 in these settings is not well established, high levels of this lectin were found in sera from lupus patients [167]. In fact, galectin-9 levels appear to be associated with kidney involvement in this disease, and a clinical trial aimed at demonstrating the clinical potential of serum galectin-9 levels in assessing disease activity and organ damage is currently active (NCT04558814).

### Concluding remarks and the future of glycomedicine

In the post-genomic area, the study of the glycome has identified key immunoregulatory pathways that integrate with canonical programs contributing to induction, amplification and resolution of innate and adaptive immune responses. Glycans and GBPs, including galectins, siglecs and C-type lectins, are central components of tolerogenic circuits that may shape both lymphoid and myeloid compartments. This effect involves several potentially overlapping mechanisms including modulation of immune checkpoint pathways, induction of tolerogenic dendritic cells, expansion of myeloid-derived suppressor cells or Tregs, control of T-cell viability and modulation of the threshold of T-cell and B-cell activation in a wide range of pathophysiologic settings. During infection, a wide range of pathogens, including viruses, bacteria, fungi and parasites, carry a particular set of glycans that are used not only to adhere and invade host cells, but also to orchestrate and tailor innate and adaptive immunity. In this regard, microbes may co-opt host glycans and GBP, engaging in a glycan mimicry process to evade and/or subvert immune responses, thus promoting infection and perpetuating the colonization of the host.

Interestingly, host cells have evolved to display a similar set of glycan structures as those used by pathogens (e.g. mannosylated, fucosylated, or sialylated structures), either to orchestrate and amplify immune responses during the development of inflammation and autoimmunity or to promote resolution of the disease and restoration of tissue homeostasis (as depicted in Fig. 2). GBPs serve to interpret this structural information, by generating different multivalent lectin-glycan structures that positively or negatively influence biologically relevant processes [168].

However, in spite of considerable progress, several questions remain unanswered and are still challenges in the field of glycoimmunology: could the glycan-GBP hub be used as a biomarker for diagnosis, prognosis and treatment decision in immune-mediated diseases? Can we interfere with glycan-GBP interactions to tailor the course of microbial infections or the inflammatory/autoimmune process? Will these interventions modulate common immune pathways leading to either immunostimulatory or immunosuppressive responses? Given their diverse glycan-binding specificities, do GBPs (C-type lectins, siglecs and galectins) cooperate, synergize or counteract each other in the control of host-pathogen interactions, immune evasion mechanisms or resolution of inflammatory responses? Can we discriminate at the structural and single cell level the contribution of GBP-glycan interactions to amplification or inhibition of inflammatory responses?

Further studies including international collaborative efforts are warranted to move forward this exciting field with particular emphasis in understanding the biology that underlies the association of glycans and GBPs, in a non-reductionist and integrative fashion, in order to capitalize this information for therapeutic and prophylactic purposes in a broad setting of immune-mediated diseases that span from infection, inflammation, autoimmunity and cancer.

## References

[1] Alves I, Fernandes Â, Santos-Pereira B, Azevedo CM, Pinho SS (2022). Glycans as a key factor in self and nonself discrimination: impact on the breach of immune tolerance. FEBS Lett..

[2] Mariño KV, Cagnoni AJ, Croci DO, Rabinovich GA (2023). Targeting galectin-driven regulatory circuits in cancer and fibrosis. Nat Rev Drug Discov..

[3] Ohtsubo K, Marth JD (2006). Glycosylation in cellular mechanisms of health and disease. Cell..

[4] Pinho SS, Reis CA (2015). Glycosylation in cancer: mechanisms and clinical implications. Nat Rev Cancer..

[5] Rabinovich GA, Croci DO (2012). Regulatory circuits mediated by lectin-glycan interactions in autoimmunity and cancer. Immunity..

[6] Rabinovich GA, Toscano MA (2009). Turning ‘sweet’ on immunity: galectin–glycan interactions in immune tolerance and inflammation. Nat Rev Immunol..

[7] Verhelst X, Dias AM, Colombel J-F, Vermeire S, Van Vlierberghe H, Callewaert N (2020). Protein Glycosylation as a Diagnostic and Prognostic Marker of Chronic Inflammatory Gastrointestinal and Liver Diseases. Gastroenterology..

[8] Crispin M, Ward AB, Wilson IA (2018). Structure and immune recognition of the HIV glycan shield. Annu Rev Biophys..

[9] Krumm SA, Doores KJ (2020). Targeting glycans on human pathogens for vaccine design. Curr Top Microbiol Immunol..

[10] Varki A (2006). Nothing in glycobiology makes sense, except in the light of evolution. Cell..

[11] Varki A (2011). Since there are PAMPs and DAMPs, there must be SAMPs? Glycan “self-associated molecular patterns” dampen innate immunity, but pathogens can mimic them. Glycobiology..

[12] National Research Council (US) Committee on Assessing the Importance andImpact of Glycomics and Glycosciences. Transforming glycoscience: A roadmap for the future. 2012. 10.17226/13446.

[13] Rudd PM, Elliott T, Cresswell P, Wilson IA, Dwek RA (2001). Glycosylation and the immune system. Science..

[14] Reily C, Stewart TJ, Renfrow MB, Novak J (2019). Glycosylation in health and disease. Nat Rev Nephrol..

[15] Moremen KW, Tiemeyer M, Nairn AV (2012). Vertebrate protein glycosylation: diversity, synthesis and function. Nat Rev Mol Cell Biol..

[16] Varki A, Cummings RD, Esko JD, Freeze HH, Stanley P, Bertozzi CR, et al. Essentials of glycobiology. New York, USA: Cold Spring Harbor Laboratory Press; 2009. 20301239

[17] Cummings RD (2009). The repertoire of glycan determinants in the human glycome. Mol Biosyst..

[18] Gabius H-J, André S, Jiménez-Barbero J, Romero A, Solís D (2011). From lectin structure to functional glycomics: principles of the sugar code. Trends Biochem Sci..

[19] Varki A, Cummings RD, Esko JD, Stanley P, Hart GW, Aebi M. Essentials of glycobiology (Cold Spring Harbor Laboratory Press; 2022. 10.1101/9781621824213. 35536922

[20] Lau KS, Partridge EA, Grigorian A, Silvescu CI, Reinhold VN, Demetriou M (2007). Complex N-glycan number and degree of branching cooperate to regulate cell proliferation and differentiation. Cell..

[21] Medina-Cano D, Ucuncu E, Nguyen LS, Nicouleau M, Lipecka J, Bizot J-C (2018). High N-glycan multiplicity is critical for neuronal adhesion and sensitizes the developing cerebellum to N-glycosylation defect. Elife..

[22] Dias AM, Correia A, Pereira MS, Almeida CR, Alves I, Pinto V (2018). Metabolic control of T cell immune response through glycans in inflammatory bowel disease. Proc Natl Acad Sci USA..

[23] Dias AM, Dourado J, Lago P, Cabral J, Marcos-Pinto R, Salgueiro P (2014). Dysregulation of T cell receptor N-glycosylation: a molecular mechanism involved in ulcerative colitis. Hum Mol Genet..

[24] Dennis JW, Warren CE, Granovsky M, Demetriou M (2001). Genetic defects in N-glycosylation and cellular diversity in mammals. Curr Opin Struct Biol..

[25] Morosi LG, Cutine AM, Cagnoni AJ, Manselle-Cocco MN, Croci DO, Merlo JP, et al. Control of intestinal inflammation by glycosylation-dependent lectin-driven immunoregulatory circuits. Sci Adv. 2021; 7. 10.1126/sciadv.abf8630 PMC8213219 34144987

[26] Suzuki R, Nakamura Y, Koiwai R, Fuseya S, Murakami Y, Hagiwara K, et al. Global Loss of Core 1-Derived O-Glycans in Mice Leads to High Mortality Due to Acute Kidney Failure and Gastric Ulcers. Int J Mol Sci. 2022; 23. 10.3390/ijms23031273 PMC8835874 35163200

[27] Fu J, Wei B, Wen T, Johansson MEV, Liu X, Bradford E (2011). Loss of intestinal core 1-derived O-glycans causes spontaneous colitis in mice. J Clin Invest..

[28] Liu F, Fu J, Bergstrom K, Shan X, McDaniel JM, McGee S, et al. Core 1-derived mucin-type O-glycosylation protects against spontaneous gastritis and gastric cancer. J Exp Med. 2020; 217. 10.1084/jem.20182325 PMC7037257 31645367

[29] Marth JD, Grewal PK (2008). Mammalian glycosylation in immunity. Nat Rev Immunol..

[30] Ryan SO, Cobb BA (2012). Roles for major histocompatibility complex glycosylation in immune function. Semin Immunopathol..

[31] Wolfert MA, Boons G-J (2013). Adaptive immune activation: glycosylation does matter. Nat Chem Biol..

[32] Demetriou M, Granovsky M, Quaggin S, Dennis JW (2001). Negative regulation of T-cell activation and autoimmunity by Mgat5 N-glycosylation. Nature..

[33] Grigorian A, Torossian S, Demetriou M (2009). T-cell growth, cell surface organization, and the galectin-glycoprotein lattice. Immunol Rev..

[34] Johnson JL, Jones MB, Ryan SO, Cobb BA (2013). The regulatory power of glycans and their binding partners in immunity. Trends Immunol..

[35] Larsen MD, de Graaf EL, Sonneveld ME, Plomp HR, Nouta J, Hoepel W (2021). Afucosylated IgG characterizes enveloped viral responses and correlates with COVID-19 severity. Science..

[36] Vicente MM, Alves I, Gaifem J, Rodrigues CS, Fernandes Â, Dias AM (2022). Altered IgG glycosylation at COVID-19 diagnosis predicts disease severity. Eur J Immunol..

[37] van Kooyk Y, Rabinovich GA (2008). Protein-glycan interactions in the control of innate and adaptive immune responses. Nat Immunol..

[38] Schnaar RL (2015). Glycans and glycan-binding proteins in immune regulation: a concise introduction to glycobiology for the allergist. J Allergy Clin Immunol..

[39] Geijtenbeek TBH, Gringhuis SI (2009). Signalling through C-type lectin receptors: shaping immune responses. Nat Rev Immunol..

[40] Quintana JI, Atxabal U, Unione L, Ardá A, Jiménez-Barbero J (2023). Exploring multivalent carbohydrate-protein interactions by NMR. Chem Soc Rev.

[41] Svajger U, Anderluh M, Jeras M, Obermajer N (2010). C-type lectin DC-SIGN: an adhesion, signalling and antigen-uptake molecule that guides dendritic cells in immunity. Cell Signal..

[42] Osorio F (2011). Reis e Sousa C. Myeloid C-type lectin receptors in pathogen recognition and host defense. Immunity..

[43] Fuertes MB, Molinero LL, Toscano MA, Ilarregui JM, Rubinstein N, Fainboim L (2004). Regulated expression of galectin-1 during T-cell activation involves Lck and Fyn kinases and signaling through MEK1/ERK, p38 MAP kinase and p70S6 kinase. Mol Cell Biochem..

[44] Toscano MA, Bianco GA, Ilarregui JM, Croci DO, Correale J, Hernandez JD (2007). Differential glycosylation of TH1, TH2 and TH-17 effector cells selectively regulates susceptibility to cell death. Nat Immunol..

[45] Santucci L, Fiorucci S, Rubinstein N, Mencarelli A, Palazzetti B, Federici B (2003). Galectin-1 suppresses experimental colitis in mice. Gastroenterology..

[46] Mourcin F, Breton C, Tellier J, Narang P, Chasson L, Jorquera A (2011). Galectin-1–expressing stromal cells constitute a specific niche for pre-BII cell development in mouse bone marrow. Blood..

[47] Smith LK, Fawaz K, Treanor B (2021). Galectin-9 regulates the threshold of B cell activation and autoimmunity. Elife..

[48] Mortales C-L, Lee S-U, Demetriou M (2020). N-glycan branching is required for development of mature B cells. J Immunol..

[49] Starossom SC (2012). Galectin-1 deactivates classically activated microglia and protects from inflammation-induced neurodegeneration. Immunity..

[50] Ilarregui JM, Croci DO, Bianco GA, Toscano MA, Salatino M, Vermeulen ME (2009). Tolerogenic signals delivered by dendritic cells to T cells through a galectin-1-driven immunoregulatory circuit involving interleukin 27 and interleukin 10. Nat Immunol..

[51] Dardalhon V, Anderson AC, Karman J, Apetoh L, Chandwaskar R, Lee DH (2010). Tim-3/galectin-9 pathway: regulation of Th1 immunity through promotion of CD11b+Ly-6G+ myeloid cells. J Immunol..

[52] Liu F-T, Stowell SR. The role of galectins in immunity and infection. Nat. Rev. Immunol. 2023, 1–16. 10.1038/s41577-022-00829-7. 10.1038/s41577-022-00829-7 PMC9842223 36646848

[53] Russo AJ, Vasudevan SO, Méndez-Huergo SP, Kumari P, Menoret A, Duduskar S (2021). Intracellular immune sensing promotes inflammation via gasdermin D-driven release of a lectin alarmin. Nat Immunol..

[54] Liu F-T, Rabinovich GA (2010). Galectins: regulators of acute and chronic inflammation. Ann N Y Acad Sci..

[55] Liu F-T, Hsu DK (2007). The role of galectin-3 in promotion of the inflammatory response. Drug N Perspect..

[56] Hokama A, Mizoguchi E, Sugimoto K, Shimomura Y, Tanaka Y, Yoshida M (2004). Induced reactivity of intestinal CD4(+) T cells with an epithelial cell lectin, galectin-4, contributes to exacerbation of intestinal inflammation. Immunity..

[57] Hong M-H, Weng I-C, Li F-Y, Lin W-H, Liu F-T (2021). Intracellular galectins sense cytosolically exposed glycans as danger and mediate cellular responses. J Biomed Sci..

[58] Weng I-C, Chen H-L, Lo T-H, Lin W-H, Chen H-Y, Hsu DK (2018). Cytosolic galectin-3 and -8 regulate antibacterial autophagy through differential recognition of host glycans on damaged phagosomes. Glycobiology..

[59] Thurston TLM, Wandel MP, von Muhlinen N, Foeglein A, Randow F (2012). Galectin 8 targets damaged vesicles for autophagy to defend cells against bacterial invasion. Nature..

[60] Liu F-T, Patterson RJ, Wang JL (2002). Intracellular functions of galectins. Biochim Biophys Acta..

[61] Crocker PR, Paulson JC, Varki A (2007). Siglecs and their roles in the immune system. Nat Rev Immunol..

[62] Chang Y-C, Nizet V (2014). The interplay between Siglecs and sialylated pathogens. Glycobiology..

[63] Han S, Collins BE, Bengtson P, Paulson JC (2005). Homomultimeric complexes of CD22 in B cells revealed by protein-glycan cross-linking. Nat Chem Biol..

[64] Chang Y-C, Olson J, Beasley FC, Tung C, Zhang J, Crocker PR (2014). Group B Streptococcus engages an inhibitory Siglec through sialic acid mimicry to blunt innate immune and inflammatory responses in vivo. PLoS Pathog..

[65] Secundino I, Lizcano A, Roupé KM, Wang X, Cole JN, Olson J (2016). Host and pathogen hyaluronan signal through human siglec-9 to suppress neutrophil activation. J Mol Med..

[66] Kaneko Y, Nimmerjahn F, Ravetch JV (2006). Anti-inflammatory activity of immunoglobulin G resulting from Fc sialylation. Science..

[67] Anthony RM, Kobayashi T, Wermeling F, Ravetch JV (2011). Intravenous gammaglobulin suppresses inflammation through a novel T(H)2 pathway. Nature..

[68] Bayry J, Bansal K, Kazatchkine MD, Kaveri SV (2009). DC-SIGN and alpha2,6-sialylated IgG Fc interaction is dispensable for the anti-inflammatory activity of IVIg on human dendritic cells. PNAS USA..

[69] Heyl KA, Karsten CM, Slevogt H (2016). Galectin-3 binds highly galactosylated IgG1 and is crucial for the IgG1 complex mediated inhibition of C5aReceptor induced immune responses. Biochem Biophys Res Commun..

[70] Karsten CM, Pandey MK, Figge J, Kilchenstein R, Taylor PR, Rosas M (2012). Anti-inflammatory activity of IgG1 mediated by Fc galactosylation and association of FcγRIIB and dectin-1. Nat Med..

[71] Šimurina M, de Haan N, Vučković F, Kennedy NA, Štambuk J, Falck D (2018). Glycosylation of Immunoglobulin G Associates With Clinical Features of Inflammatory Bowel Diseases. Gastroenterology..

[72] Parekh RB, Dwek RA, Sutton BJ, Fernandes DL, Leung A, Stanworth D (1985). Association of rheumatoid arthritis and primary osteoarthritis with changes in the glycosylation pattern of total serum IgG. Nature..

[73] Parekh RB, Roitt IM, Isenberg DA, Dwek RA, Ansell BM, Rademacher TW (1988). Galactosylation of IgG associated oligosaccharides: reduction in patients with adult and juvenile onset rheumatoid arthritis and relation to disease activity. Lancet..

[74] Malhotra R, Wormald MR, Rudd PM, Fischer PB, Dwek RA, Sim RB (1995). Glycosylation changes of IgG associated with rheumatoid arthritis can activate complement via the mannose-binding protein. Nat Med..

[75] Shade K-TC, Platzer B, Washburn N, Mani V, Bartsch YC, Conroy M (2015). A single glycan on IgE is indispensable for initiation of anaphylaxis. J Exp Med..

[76] Dube DH, Bertozzi CR (2005). Glycans in cancer and inflammation–potential for therapeutics and diagnostics. Nat Rev Drug Discov..

[77] Lujan AL, Croci DO, Rabinovich GA, Damiani MT (2022). Galectins as potential therapeutic targets in STIs in the female genital tract. Nat Rev Urol..

[78] Koropatkin NM, Cameron EA, Martens EC (2012). How glycan metabolism shapes the human gut microbiota. Nat Rev Microbiol..

[79] Cambi A, Netea MG, Mora-Montes HM, Gow NAR, Hato SV, Lowman DW (2008). Dendritic cell interaction with *Candida albicans* critically depends on N-linked mannan. J Biol Chem..

[80] Bates S, Hughes HB, Munro CA, Thomas WPH, MacCallum DM, Bertram G (2006). Outer chain N-glycans are required for cell wall integrity and virulence of Candida albicans. J Biol Chem..

[81] Munro CA, Bates S, Buurman ET, Hughes HB, Maccallum DM, Bertram G (2005). Mnt1p and Mnt2p of *Candida albicans* are partially redundant alpha-1,2-mannosyltransferases that participate in O-linked mannosylation and are required for adhesion and virulence. J Biol Chem..

[82] Gow NAR, van de Veerdonk FL, Brown AJP, Netea MG (2011). Candida albicans morphogenesis and host defence: discriminating invasion from colonization. Nat Rev Microbiol..

[83] Ramirez-Ortiz ZG, Means TK (2012). The role of dendritic cells in the innate recognition of pathogenic fungi (*A. fumigatus, C. neoformans* and *C. albicans*). Virulence..

[84] Gringhuis SI, den Dunnen J, Litjens M, van Het Hof B, van Kooyk Y, Geijtenbeek TBH (2007). C-type lectin DC-SIGN modulates Toll-like receptor signaling via Raf-1 kinase-dependent acetylation of transcription factor NF-kappaB. Immunity..

[85] van de Veerdonk FL, Marijnissen RJ, Kullberg BJ, Koenen HJPM, Cheng S-C, Joosten I (2009). The macrophage mannose receptor induces IL-17 in response to Candida albicans. Cell Host Microbe..

[86] Brown GD, Herre J, Williams DL, Willment JA, Marshall ASJ, Gordon S (2003). Dectin-1 mediates the biological effects of beta-glucans. J Exp Med..

[87] Gantner BN, Simmons RM, Canavera SJ, Akira S, Underhill DM (2003). Collaborative induction of inflammatory responses by dectin-1 and Toll-like receptor 2. J Exp Med..

[88] Sato K, Yang X, Yudate T, Chung J-S, Wu J, Luby-Phelps K (2006). Dectin-2 is a pattern recognition receptor for fungi that couples with the Fc receptor gamma chain to induce innate immune responses. J Biol Chem..

[89] Robinson MJ, Osorio F, Rosas M, Freitas RP, Schweighoffer E, Gross O (2009). Dectin-2 is a Syk-coupled pattern recognition receptor crucial for Th17 responses to fungal infection. J Exp Med..

[90] Kohatsu L, Hsu DK, Jegalian AG, Liu F-T, Baum LG (2006). Galectin-3 induces death of Candida species expressing specific beta-1,2-linked mannans. J Immunol..

[91] Fradin C, Poulain D, Jouault T (2000). beta-1,2-linked oligomannosides from Candida albicans bind to a 32-kilodalton macrophage membrane protein homologous to the mammalian lectin galectin-3. Infect Immun..

[92] Watanabe Y, Bowden TA, Wilson IA, Crispin M (2019). Exploitation of glycosylation in enveloped virus pathobiology. Biochim Biophys acta Gen Subj..

[93] Scanlan CN, Offer J, Zitzmann N, Dwek RA (2007). Exploiting the defensive sugars of HIV-1 for drug and vaccine design. Nature..

[94] Behrens A-J, Vasiljevic S, Pritchard LK, Harvey DJ, Andev RS, Krumm SA (2016). Composition and antigenic effects of individual glycan sites of a trimeric HIV-1 envelope glycoprotein. Cell Rep..

[95] Kong L, Lee JH, Doores KJ, Murin CD, Julien J-P, McBride R (2013). Supersite of immune vulnerability on the glycosylated face of HIV-1 envelope glycoprotein gp120. Nat Struct Mol Biol..

[96] Watanabe Y, Berndsen ZT, Raghwani J, Seabright GE, Allen JD, Pybus OG (2020). Vulnerabilities in coronavirus glycan shields despite extensive glycosylation. Nat Commun..

[97] Walls AC, Tortorici MA, Frenz B, Snijder J, Li W, Rey FA (2016). Glycan shield and epitope masking of a coronavirus spike protein observed by cryo-electron microscopy. Nat Struct Mol Biol..

[98] Amraei R, Yin W, Napoleon MA, Suder EL, Berrigan J, Zhao Q (2021). CD209L/L-SIGN and CD209/DC-SIGN act as receptors for SARS-CoV-2. ACS Cent Sci..

[99] Cramer J, Lakkaichi A, Aliu B, Jakob RP, Klein S, Cattaneo I (2021). Sweet drugs for bad bugs: a glycomimetic strategy against the DC-SIGN-mediated dissemination of SARS-CoV-2. J Am Chem Soc..

[100] Thépaut M, Luczkowiak J, Vivès C, Labiod N, Bally I, Lasala F (2021). DC/L-SIGN recognition of spike glycoprotein promotes SARS-CoV-2 trans-infection and can be inhibited by a glycomimetic antagonist. PLoS Pathog..

[101] Gao C, Zeng J, Jia N, Stavenhagen K, Matsumoto Y, Zhang H, et al. SARS-CoV-2 spike protein interacts with multiple innate immune receptors. bioRxiv: the preprint server for biology. 2020. 10.1101/2020.07.29.227462

[102] Cai G, Du M, Bossé Y, Albrecht H, Qin F, Luo X (2021). SARS-CoV-2 impairs dendritic cells and regulates DC-SIGN gene expression in tissues. Int J Mol Sci..

[103] Logan SM (2006). Flagellar glycosylation - a new component of the motility repertoire?. Microbiology..

[104] Charbonneau M-E, Girard V, Nikolakakis A, Campos M, Berthiaume F, Dumas F (2007). O-linked glycosylation ensures the normal conformation of the autotransporter adhesin involved in diffuse adherence. J Bacteriol..

[105] Nandakumar S, Kannanganat S, Dobos KM, Lucas M, Spencer JS, Fang S (2013). O-mannosylation of the *Mycobacterium tuberculosis* adhesin Apa is crucial for T cell antigenicity during infection but is expendable for protection. PLoS Pathog..

[106] Alderwick LJ, Harrison J, Lloyd GS, Birch HL (2015). The mycobacterial cell wall–peptidoglycan and arabinogalactan. Cold Spring Harb Perspect Med..

[107] Vergne I, Gilleron M, Nigou J (2014). Manipulation of the endocytic pathway and phagocyte functions by *Mycobacterium tuberculosis* lipoarabinomannan. Front. Cell Infect Microbiol..

[108] Kleinnijenhuis J, Oosting M, Joosten LAB, Netea MG, Van Crevel R. Innate immune recognition of *Mycobacterium tuberculosis*. Clin Dev Immunol. 2011:405310. 10.1155/2011/405310 PMC3095423 21603213

[109] Geijtenbeek TBH, Van Vliet SJ, Koppel EA, Sanchez-Hernandez M, Vandenbroucke-Grauls CMJE, Appelmelk B (2003). Mycobacteria target DC-SIGN to suppress dendritic. cell function. J Exp Med..

[110] Yonekawa A, Saijo S, Hoshino Y, Miyake Y, Ishikawa E, Suzukawa M (2014). Dectin-2 is a direct receptor for mannose-capped lipoarabinomannan of mycobacteria. Immunity..

[111] Richmond JM, Lee J, Green DS, Kornfeld H, Cruikshank WW (2012). Mannose-capped lipoarabinomannan from *Mycobacterium tuberculosis* preferentially inhibits sphingosine-1-phosphate-induced migration of Th1 cells. J Immunol..

[112] Liu C-F, Tonini L, Malaga W, Beau M, Stella A, Bouyssié D (2013). Bacterial protein-O-mannosylating enzyme is crucial for virulence of *Mycobacterium tuberculosis*. Proc Natl Acad Sci USA..

[113] Jia L, Sha S, Yang S, Taj A, Ma Y (2021). Effect of protein O-mannosyltransferase (MSMEG_5447) on *M. smegmatis* and its survival in macrophages. Front Microbiol..

[114] Varki A, Gagneux P (2012). Multifarious roles of sialic acids in immunity. Ann N Y Acad Sci.

[115] Gulati S, Schoenhofen IC, Whitfield DM, Cox AD, Li J, St Michael F (2015). Utilizing CMP-sialic acid analogs to unravel neisseria gonorrhoeae lipooligosaccharide-mediated complement resistance and design novel therapeutics. PLoS Pathog..

[116] Jennings MP, Day CJ, Atack JM (2022). How bacteria utilize sialic acid during interactions with the host: snip, snatch, dispatch, match and attach. Microbiology..

[117] Stowell SR, Arthur CM, Dias-Baruffi M, Rodrigues LC, Gourdine J-P, Heimburg-Molinaro J (2010). Innate immune lectins kill bacteria expressing blood group antigen. Nat Med..

[118] Li C-S, Lo T-H, Tu T-J, Chueh D-Y, Yao C-I, Lin C-H (2023). Cytosolic galectin-4 enchains bacteria, restricts their motility, and promotes inflammasome activation in intestinal epithelial cells. Proc Natl Acad Sci USA..

[119] Wu S-C, Ho AD, Kamili NA, Wang J, Murdock KL, Cummings RD (2021). Full-length Galectin-3 is required for high affinity microbial interactions and antimicrobial activity. Front Microbiol..

[120] Lujan AL, Croci DO, Gambarte Tudela JA, Losinno AD, Cagnoni AJ, Mariño KV (2018). Glycosylation-dependent galectin-receptor interactions promote *Chlamydia trachomatis* infection. Proc Natl Acad Sci USA.

[121] Okumura CYM, Baum LG, Johnson PJ (2008). Galectin-1 on cervical epithelial cells is a receptor for the sexually transmitted human parasite *Trichomonas vaginalis*. Cell Microbiol.

[122] Poncini CV, Ilarregui JM, Batalla EI, Engels S, Cerliani JP, Cucher MA (2015). *Trypanosoma cruzi* infection imparts a regulatory program in dendritic cells and T cells via galectin-1-dependent mechanisms. J Immunol..

[123] Davicino RC, Méndez-Huergo SP, Eliçabe RJ, Stupirski JC, Autenrieth I, Di Genaro MS (2017). Galectin-1-driven tolerogenic programs aggravate yersinia enterocolitica infection by repressing antibacterial immunity. J Immunol.

[124] Rubione J, Pérez PS, Czernikier A, Duette GA, Pereyra Gerber FP, Salido J (2022). A dynamic interplay of circulating extracellular vesicles and galectin-1 reprograms viral latency during HIV-1. Infect MBio..

[125] Abdel-Mohsen M, Chavez L, Tandon R, Chew GM, Deng X, Danesh A (2016). Human Galectin-9 is a potent mediator of HIV transcription and reactivation. PLoS Pathog..

[126] Levroney EL, Aguilar HC, Fulcher JA, Kohatsu L, Pace KE, Pang M (2005). Novel innate immune functions for galectin-1: galectin-1 inhibits cell fusion by Nipah virus envelope glycoproteins and augments dendritic cell secretion of proinflammatory cytokines. J Immunol..

[127] Wacker M, Linton D, Hitchen PG, Nita-Lazar M, Haslam SM, North SJ (2002). N-linked glycosylation in *Campylobacter jejuni* and its functional transfer into. E. coli. Science..

[128] Alemka A, Nothaft H, Zheng J, Szymanski CM (2013). N-glycosylation of *Campylobacter jejuni* surface proteins promotes bacterial fitness. Infect Immun..

[129] Szymanski CM, Burr DH, Guerry P (2002). Campylobacter protein glycosylation affects host cell interactions. Infect Immun..

[130] van Sorge NM, Bleumink NMC, van Vliet SJ, Saeland E, van der Pol W-L, van Kooyk Y (2009). N-glycosylated proteins and distinct lipooligosaccharide glycoforms of Campylobacter jejuni target the human C-type lectin receptor MGL. Cell Microbiol..

[131] Jostins L, Ripke S, Weersma RK, Duerr RH, McGovern DP, Hui KY (2012). Host-microbe interactions have shaped the genetic architecture of inflammatory bowel disease. Nature..

[132] Robinson BS, Saeedi B, Arthur CM, Owens J, Naudin C, Ahmed N (2020). Galectin-9 is a novel regulator of epithelial restitution. Am J Pathol..

[133] McGovern DPB, Jones MR, Taylor KD, Marciante K, Yan X, Dubinsky M (2010). Fucosyltransferase 2 (FUT2) non-secretor status is associated with Crohn’s disease. Hum Mol Genet..

[134] Goto Y, Obata T, Kunisawa J, Sato S, Ivanov II, Lamichhane A (2014). Innate lymphoid cells regulate intestinal epithelial cell glycosylation. Science..

[135] Coyne MJ, Reinap B, Lee MM, Comstock LE (2005). Human symbionts use a host-like pathway for surface fucosylation. Science..

[136] Taleb V, Liao Q, Narimatsu Y, García-García A, Compañón I, Borges RJ (2022). Structural and mechanistic insights into the cleavage of clustered O-glycan patches-containing glycoproteins by mucinases of the human gut. Nat Commun..

[137] Hayase E, Hayase T, Jamal MA, Miyama T, Chang C-C, Ortega MR (2022). Mucus-degrading Bacteroides link carbapenems to aggravated graft-versus-host disease. Cell..

[138] Zeng J-Q, Xu C-D, Zhou T, Wu J, Lin K, Liu W (2015). Enterocyte dendritic cell-specific intercellular adhesion molecule-3-grabbing non-integrin expression in inflammatory bowel disease. World J Gastroenterol..

[139] Iliev ID, Funari VA, Taylor KD, Nguyen Q, Reyes CN, Strom SP (2012). Interactions between commensal fungi and the C-type lectin receptor Dectin-1 influence colitis. Science..

[140] Yang WH, Westman JS, Heithoff DM, Sperandio M, Cho JW, Mahan MJ (2021). Neu3 neuraminidase induction triggers intestinal inflammation and colitis in a model of recurrent human food-poisoning. Proc Natl Acad Sci USA..

[141] Matsuzwa M, Ota H, Hayama M, Zhang MX, Sano K, Honda T (2003). Helicobacter pylori infection up-regulates gland mucous cell-type mucins in gastric pyloric mucosa. Helicobacter..

[142] Pereira MS, Alves I, Vicente M, Campar A, Silva MC, Padrão NA (2018). Glycans as key checkpoints of T cell activity and function. Front Immunol..

[143] Mortales C-L, Lee S-U, Manousadjian A, Hayama KL, Demetriou M (2020). N-glycan branching decouples B cell innate and adaptive immunity to control inflammatory demyelination. iScience..

[144] Jiang H-R, Al Rasebi Z, Mensah-Brown E, Shahin A, Xu D, Goodyear CS (2009). Galectin-3 deficiency reduces the severity of experimental autoimmune encephalomyelitis. J Immunol..

[145] Hu Y, Yéléhé-Okouma M, Ea H-K, Jouzeau J-Y, Reboul P (2017). Galectin-3: a key player in arthritis. J Bone Spine..

[146] Wang Y, Khan A, Antonopoulos A, Bouché L, Buckley CD, Filer A (2021). Loss of α2-6 sialylation promotes the transformation of synovial fibroblasts into a pro-inflammatory phenotype in arthritis. Nat Commun..

[147] Xu D, Cui Z, Liu W, Tao R, Tao T, Shen A (2011). Tumor necrosis factor-α up-regulates the expression of β1,4-galactosyltransferase-I in human fibroblast-like synoviocytes. Inflammation..

[148] Rabinovich GA, Daly G, Dreja H, Tailor H, Riera CM, Hirabayashi J (1999). Recombinant galectin-1 and its genetic delivery suppress collagen-induced arthritis via T cell apoptosis. J Exp Med..

[149] Mendez-Huergo SP, Hockl PF, Stupirski JC, Maller SM, Morosi LG, Pinto NA (2018). Clinical relevance of Galectin-1 and Galectin-3 in rheumatoid arthritis patients: differential regulation and correlation with disease activity. Front Immunol..

[150] Pagan JD, Kitaoka M, Anthony RM (2018). Engineered sialylation of pathogenic antibodies in vivo attenuates autoimmune disease. Cell..

[151] Smyth DJ, Cooper JD, Howson JMM, Clarke P, Downes K, Mistry T (2011). FUT2 nonsecretor status links type 1 diabetes susceptibility and resistance to infection. Diabetes..

[152] Zhao S-Q, Hu Z (2016). Mannose-binding lectin and diabetic nephropathy in type 1 diabetes. J Clin Lab Anal..

[153] Axelgaard E, Østergaard JA, Thiel S, Hansen TK (2017). Diabetes is associated with increased autoreactivity of mannan-binding lectin. J Diabetes Res..

[154] Perone MJ, Bertera S, Shufesky WJ, Divito SJ, Montecalvo A, Mathers AR (2009). Suppression of autoimmune diabetes by soluble galectin-1. J Immunol..

[155] Sundblad V, Garcia-Tornadu IA, Ornstein AM, Martínez Allo VC, Lorenzo R, Gatto SG (2021). Galectin-1 impacts on glucose homeostasis by modulating pancreatic insulin release. Glycobiology..

[156] Alves I, Santos-Pereira B, Dalebout H, Santos S, Vicente MM, Campar A (2021). Protein mannosylation as a diagnostic and prognostic biomarker of lupus nephritis: an unusual glycan neoepitope in systemic lupus erythematosus. Arthritis Rheumatol..

[157] Urushihara M, Kagami S, Yasutomo K, Ito M, Kondo S, Kitamura A (2004). Sisters with alpha-mannosidosis and systemic lupus erythematosus. Eur J Pediatr..

[158] Chui D, Sellakumar G, Green R, Sutton-Smith M, McQuistan T, Marek K (2001). Genetic remodeling of protein glycosylation in vivo induces autoimmune disease. Proc Natl Acad Sci USA..

[159] Green RS, Stone EL, Tenno M, Lehtonen E, Farquhar MG, Marth JD (2007). Mammalian N-glycan branching protects against innate immune self-recognition and inflammation in autoimmune disease pathogenesis. Immunity..

[160] Hashii N, Kawasaki N, Itoh S, Nakajima Y, Kawanishi T, Yamaguchi T (2009). Alteration of N-glycosylation in the kidney in a mouse model of systemic lupus erythematosus: relative quantification of N-glycans using an isotope-tagging method. Immunology..

[161] Cai M, Zhou T, Wang X, Shang M, Zhang Y, Luo M (2016). DC-SIGN expression on podocytes and its role in inflammatory immune response of lupus nephritis. Clin Exp Immunol..

[162] Lhotta K, Würzner R, König P (1999). Glomerular deposition of mannose-binding lectin in human glomerulonephritis. Nephrol Dial Transplant..

[163] Szabó E, Hornung Á, Monostori É, Bocskai M, Czibula Á, Kovács L (2019). Altered cell surface N-glycosylation of resting and activated T cells in systemic lupus erythematosus. Int J Mol Sci..

[164] Rabinovich GA, Modesti NM, Castagna LF, Landa CA, Riera CM, Sotomayor CE (1997). Specific inhibition of lymphocyte proliferation and induction of apoptosis by CLL-I, a beta-galactoside-binding lectin. J Biochem..

[165] Martínez Allo VC, Hauk V, Sarbia N, Pinto NA, Croci DO, Dalotto-Moreno T (2020). Suppression of age-related salivary gland autoimmunity by glycosylation-dependent galectin-1-driven immune inhibitory circuits. Proc Natl Acad Sci USA..

[166] Beccaria CG, Amezcua Vesely MC, Fiocca Vernengo F, Gehrau RC, Ramello MC, Tosello Boari J (2018). Galectin-3 deficiency drives lupus-like disease by promoting spontaneous germinal centers formation via IFN-γ. Nat Commun..

[167] Matsuoka N, Fujita Y, Temmoku J, Furuya MY, Asano T, Sato S (2020). Galectin-9 as a biomarker for disease activity in systemic lupus erythematosus. PLoS ONE..

[168] Elola MT, Blidner AG, Ferragut F, Bracalente C, Rabinovich GA (2015). Assembly, organization and regulation of cell-surface receptors by lectin-glycan complexes. Biochem J..

